# Greater Beta-Adrenergic Receptor Mediated Vasodilation in Women Using Oral Contraceptives

**DOI:** 10.3389/fphys.2016.00215

**Published:** 2016-06-08

**Authors:** Jacqueline K. Limberg, Garrett L. Peltonen, Rebecca E. Johansson, John W. Harrell, Jeremy M. Kellawan, Marlowe W. Eldridge, Joshua J. Sebranek, Benjamin J. Walker, William G. Schrage

**Affiliations:** ^1^Department of Kinesiology, University of WisconsinMadison, WI, USA; ^2^Department of Pediatrics, University of WisconsinMadison, WI, USA; ^3^Department of Anesthesiology, University of WisconsinMadison, WI, USA

**Keywords:** blood flow, isoproterenol, endothelium-dependent vasodilation, smooth muscle, neural control

## Abstract

**Background:** β-adrenergic receptors play an important role in mitigating the pressor effects of sympathetic nervous system activity in young women. Based on recent data showing oral contraceptive use in women abolishes the relationship between muscle sympathetic nervous system activity and blood pressure, we hypothesized forearm blood flow responses to a β-adrenergic receptor agonist would be greater in young women currently using oral contraceptives (OC+, *n* = 13) when compared to those not using oral contraceptives (OC–, *n* = 10).

**Methods:** Women (18–35 years) were studied during the early follicular phase of the menstrual cycle (days 1–5) or placebo phase of oral contraceptive use. Forearm blood flow (FBF, Doppler ultrasound) and mean arterial blood pressure (MAP, brachial arterial catheter) were measured at baseline and during graded brachial artery infusion of the β-adrenergic receptor agonist, Isoproterenol (ISO), as well as Acetylcholine (ACH, endothelium-dependent vasodilation) and Nitroprusside (NTP, endothelium-independent vasodilation). Forearm vascular conductance was calculated (FVC = FBF/MAP, ml/min/100 mmHg) and the rise in FVC from baseline during infusion quantified vasodilation (ΔFVC = FVC_infusion_ − FVC_baseline_).

**Results:** ISO increased FVC in both groups (*p* < 0.01) and ISO-mediated ΔFVC was greater in OC+ compared to OC– (Main effect of group, *p* = 0.02). Expressing data as FVC and FBF resulted in similar conclusions. FVC responses to both ACH and NTP were also greater in OC+ compared to OC–.

**Conclusions:** These data are the first to demonstrate greater β-adrenergic receptor-mediated vasodilation in the forearm of women currently using oral contraceptives (placebo phase) when compared to those not using oral contraceptives (early follicular phase), and suggest oral contraceptive use influences neurovascular control.

## Introduction

Approximately 80% of American women will use oral contraceptives in their lifetimes (Shufelt and Bairey Merz, 2009; Boldo and White, 2011; Maguire and Westhoff, 2011). Therefore, understanding how oral contraceptives alter β-adrenergic mediated vasodilation and influence neurovascular control holds great clinical relevance. Although oral contraceptive use has been linked to improved vascular function, including increases in endothelial dependent vasodilation (Simoncini et al., 2007; Meendering et al., 2010), there are consistent reports that women using oral contraceptive pills exhibit higher systemic blood pressure (Narkiewicz et al., 1995; Cardoso et al., 1997; Boldo and White, 2011; Brito et al., 2011; Maguire and Westhoff, 2011).

β-adrenergic receptors are known to play an important role in mitigating the pressor effects of sympathetic nervous system activity in young women (Hart et al., 2011). For example, young men exhibit a positive relationship between sympathetic nervous system activity and total peripheral resistance (Hart et al., 2011). This relationship, however, is only observed in young women following β-adrenoceptor blockade (Hart et al., 2011). With this information in mind, we sought to examine the effect of current oral contraceptive use on β-adrenergic receptor mediated vasodilation using an isolated forearm model. Based on recent data showing oral contraceptive use in women abolishes the relationship between muscle sympathetic nervous system activity and blood pressure when assessed during the early follicular/placebo pill phase (Harvey et al., 2015), we hypothesized β-adrenergic receptor mediated vasodilation would be greater in young, healthy women using oral contraceptives (placebo phase) when compared to those not currently using oral contraceptives (early follicular phase).

## Materials and methods

A retrospective analysis was conducted on data collected from 33 women who participated in two previous studies from our laboratory between May 2012 and March 2015 (Harrell et al., 2015; Limberg et al., 2016). All women were healthy, non-smokers, non-obese, were not taking any cardiovascular medications and reported being sedentary (< 60 min of aerobic exercise per week, as determined by self-report). Because the risk of adverse events with oral contraceptive use increases with age and use is not encouraged in women who are >35 years, we restricted our study participants to premenopausal women between the ages of 18 and 35 years (Harvey et al., 2015). From the 33 women initially studied, 2 women >35 years of age were excluded and 8 women taking non-oral forms of hormonal contraception (e.g., intrauterine device, transdermal patch, vaginal ring, etc.) were also excluded. Women were not pregnant (confirmed by negative pregnancy test on the study day) and were studied during the early follicular phase (day 1–5) of the menstrual cycle or placebo phase of oral contraceptive use (i.e., >24 h following the last “active” pill; type of oral contraceptive was not controlled for; see Table S1). This was confirmed by the start of menses 1–5 days before study participation. Subjects underwent a minimum 10-h fast, and refrained from exercise, non-steroidal anti-inflammatory drugs, alcohol, and caffeine for 24-h prior to the study visit. Written informed consent was obtained from all subjects. All procedures were approved by the Institutional Review Board at the University of Wisconsin—Madison and conformed to the standards set by the Declaration of Helsinki.

Forearm blood flow (artery diameter, blood velocity) was measured using Doppler ultrasound (Vivid 7, General Electric; Milwaukee, WI, USA) with the subject resting supine using methods published previously (Limberg et al., 2013; Ranadive et al., 2014; Kellawan et al., 2015). The Doppler audio information from the ultrasound was converted into a digital signal using Fast Fourier Transform (Herr et al., 2010). Beat-to-beat brachial artery blood velocity and blood pressure (Arterial catheter; Transpac IV Monitoring Kit; ICUmedical; San Clemente, CA) were obtained throughout each trial.

Microvascular function was assessed by local infusion of drugs via brachial artery catheter, a widely accepted approach (Dwivedi et al., 2005; Casey and Joyner, 2011; Casey et al., 2011; Joyner and Casey, 2015). The brachial artery catheter was placed in the antecubital fossa of the non-dominant arm (the dominant arm was studied in 1 woman in the oral contraceptive group due to variations in the branching pattern of the brachial artery in the non-dominant arm that precluded study with Doppler ultrasound). All drugs were dosed to lean forearm mass as determined by DEXA using doses similar to those published previously. Isoproterenol (Isoproterenol Hydrochloride Injection, Marathon Pharmaceuticals, LLC) was infused at four separate doses (1, 3, 6, and 12 ng/100 g lean tissue/min) to assess β-adrenergic receptor mediated vasodilation (Eisenach et al., 2006, 2014; Harvey et al., 2014). Nitroprusside (Nitropress, Hospira Inc) was infused at 3 separate doses (0.5, 1.0, and 2.0 μg/100 g lean tissue/min) to assess smooth muscle responsiveness to exogenous nitric oxide (Harrell et al., 2015). Due to the retrospective nature of the study, Acetylcholine (Michol-E, Novartis Pharmaceuticals) was infused at 3 separate doses (1, 4, and 16 μg/100 g lean tissue/min) to assess endothelial function in a sub-set of subjects (Control, *n* = 6; Oral Contraceptive, *n* = 8) (Harrell et al., 2015). Each trial (Isoproterenol, Nitroprusside, Acetylcholine) was separated by a minimum of 10 min to ensure hemodynamic variables returned to baseline levels and drug orders were randomized. Trials consisted of 2 min of resting data followed by a 3-min infusion of each drug dose (for a total of ~9–12 min per infusion trial).

Data were sampled in real time with signal-processing software (PowerLab, ADinstruments, Colorado Springs, CO), digitized, and analyzed off-line. Post-processing using PowerLab Chart5 software yielded mean blood velocity and blood pressure. Steady-state hemodynamics were measured during the last 30-s of saline and each drug dose. Diameter measurements were taken immediately before increasing dosage, were assessed off-line from B-mode images, and were taken as the median of five measurements in late diastole. Blood flow was calculated as the product of mean blood velocity (cm/s) and vessel cross sectional area (radius in cm^2^) and was reported in mL/min/100 g lean mass [(blood velocity) (cross sectional area) (60 s/min) ÷ forearm lean mass]. To account for potential changes in blood pressure and assess vasodilation, vascular conductance (mL/min/100 g/100 mmHg) was calculated [Blood flow ÷ Mean arterial blood pressure].

The primary analysis was to examine the effect of current oral contraceptive use on Isoproterenol-mediated vasodilation. The secondary analysis was to examine the effect of current oral contraceptive use on Nitroprusside- and Acetylcholine-mediated vasodilation. To account for any group differences in resting vascular conductance, the main dependent variable was a change in forearm vascular conductance (FVC) from baseline levels (ΔFVC = FVC_condition_ − FVC_rest_).

Statistical analysis was completed using SigmaStat 12.0 software (Systat Software Inc., San Jose, CA, USA). Subject characteristics were compared using a Student's unpaired *t*-test, and hemodynamic variables were analyzed using an analysis of variance approach to determine the significance of the fixed effect of group and dose, in addition to group-by-dose interactions. Student-Neuman-Keuls post hoc comparisons were performed when significant effects were observed. All data are presented as Mean ± Standard error of the mean, and significance was determined *a priori* at *p* ≤ 0.05. Sample size was determined using group differences from previously published data [minimum detectable difference in means of 1.9 mL/min/100 mL/mmHg; expected standard deviation of residuals of 1.4 mL/min/100 mL/mmHg] (Harvey et al., 2014). With these data, we determined 10 subjects would provide 80% power to detect a significant difference in forearm vascular conductance at the α = 0.05 level.

## Results

Data from 23 young, healthy women (Control, *n* = 10; Oral contraceptive, *n* = 13) were included in the current study (Table 1). Groups were not different in regard to age, height, weight, body mass index, lean forearm mass, or fasting glucose levels (*p* > 0.05). Women currently taking oral contraceptives exhibited significantly higher body fat and total cholesterol when compared to control women (Table 1; *p* = 0.01 and *p* < 0.01, respectively). In addition, mean arterial blood pressure (~2–4 mmHg) and heart rate (~5–10 beat/min) were elevated in women currently taking oral contraceptives when compared with control women (Tables 2–**4**). No group differences in brachial artery diameter were observed under any condition (Tables 2–**4**).

**Table 1:** **Subject Demographics**.

	**Control (*n* = 10)**	**Oral Contraceptive (*n* = 13)**	***p*-value**
Age (yrs)	24 ± 1	22 ± 1	0.22
Height (cm)	165 ± 2	167 ± 1	0.49
Weight (kg)	57 ± 2	62 ± 1	0.06
BMI (kg/m2)	21 ± 1	22 ± 1	0.09
Body fat (%)	29 ± 2	36 ± 1	**0.01**
Lean forearm mass (g)	555 ± 24	589 ± 19	0.27
Glucose (mg/dL)	75 ± 2	70 ± 3	0.19
Total cholesterol (mg/dL)	142 ± 8	189 ± 10	**<0.01**

*Data are presented as Mean ± SEM. BMI = body mass index. Body fat determined by DEXA. Bold values highlight significance*.

**Table 2:** **Effect of Isoproterenol in women using oral contraceptives**.

	**Control (*n* = 10)**	**Oral Contraceptive (*n* = 13)**	**Main effect of Isoproterenol**	**Main effect of Group**
**DIAMETER (cm)**				
Baseline	0.33 ± 0.01	0.33 ± 0.01	0.565	0.885
1 ng/100 g/min	0.34 ± 0.01	0.34 ± 0.01		
3 ng/100 g/min	0.34 ± 0.01	0.34 ± 0.01		
6 ng/100 g/min	0.34 ± 0.01	0.34 ± 0.01		
12 ng/100 g/min	0.34 ± 0.01	0.35 ± 0.01		
**HEART RATE (beats/min)**				
Baseline	57 ± 3	66 ± 2	0.929	**<0.001**
1 ng/100 g/min	60 ± 2	65 ± 2		
3 ng/100 g/min	59 ± 3	65 ± 2		
6 ng/100 g/min	59 ± 3	66 ± 2		
12 ng/100 g/min	59 ± 3	68 ± 2		
**MEAN ARTERIAL BLOOD PRESSURE (mmHg)**				
Baseline	83 ± 2	87 ± 2	0.0963	**0.002**
1 ng/100 g/min	83 ± 2	86 ± 2		
3 ng/100 g/min	81 ± 2	86 ± 2		
6 ng/100 g/min	82 ± 2	87 ± 2		
12 ng/100 g/min	82 ± 3	87 ± 3		
**FOREARM BLOOD FLOW (mL/min/100 g)**				
Baseline	4.9 ± 0.9	6.1 ± 0.6	**<0.001**	**<0.001**
1 ng/100 g/min	7.1 ± 0.8	9.0 ± 0.8		
3 ng/100 g/min	9.3 ± 0.9^A^	13.0 ± 1.1^A^		
6 ng/100 g/min	13.4 ± 1.8^A^^B^^C^	17.7 ± 1.8^A^^B^^C^		
12 ng/100 g/min	16.0 ± 1.7^A^^B^^C^	21.6 ± 1.9^A^^B^^C^		
**FOREARM VASCULAR CONDUCTANCE (mL/min/100 g/100 mmHg)**				
Baseline	5.8 ± 1.0	7.1 ± 0.7	**<0.001**	**0.002**
1 ng/100 g/min	8.4 ± 0.8	10.6 ± 1.1		
3 ng/100 g/min	11.4 ± 0.9^A^	15.5 ± 1.6^A^		
6 ng/100 g/min	16.4 ± 2.2^A^^B^^C^	20.9 ± 2.6^A^^B^^C^		
12 ng/100 g/min	19.3 ± 1.7^A^^B^^C^	25.5 ± 2.7^A^^B^^C^		

*Data are presented as Mean ± SEM*.

A p < 0.05 vs. Baseline;

B p < 0.05 vs. 1 ng/100 g/min;

C *p < 0.05 vs. 3 ng/100 g/min. From the initial model which included Group-by-Isoproterenol interaction effect, no significant interaction was detected (p > 0.05). Bold values highlight significance*.

**Figure 1:**
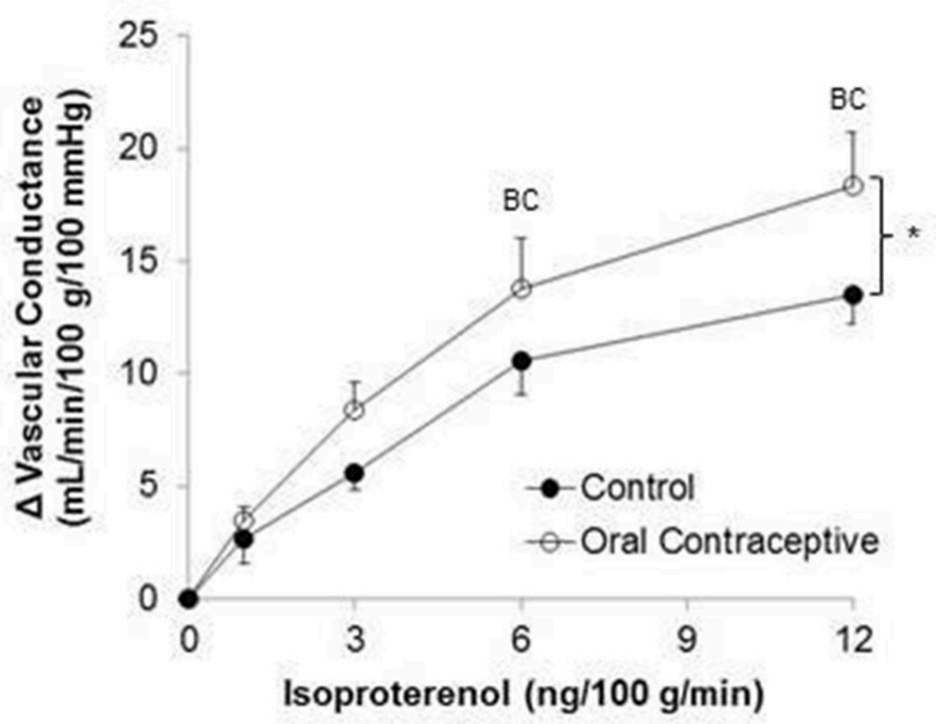
**Effect of Isoproterenol on forearm vascular conductance in women using oral contraceptives**. (Control, *n* = 10; Oral contraceptive, *n* = 13) Isoproterenol infusion resulted in a dose-dependent increase in vascular conductance in both groups (*p* < 0.001). The rise in vascular conductance from baseline with Isoproterenol infusion was greater in women taking oral contraceptives when compared to controls (*p* = 0.012). Data are presented as Mean ± SEM. ^*^p < 0.05 vs. Control; ^B^*p* < 0.05 vs. 1 ng/100 g/min; ^C^*p* < 0.05 vs. 3 ng/100 g/min.

Isoproterenol infusion (Control, *n* = 10; Oral contraceptive, *n* = 13) resulted in a dose-dependent increase in vascular conductance (Table 2, Figure 1; Main effect of ISO *p* < 0.001). Isoproterenol-mediated vasodilation was greater in women taking oral contraceptives when compared to control women (Figure 1; Main effect of group, *p* = 0.002). Similarly, the rise (Δ) in vascular conductance from baseline with Isoproterenol infusion was greater in women taking oral contraceptives when compared to controls (Figure 1; Main effect of group, *p* = 0.012). No group-by-dose interactions were observed. Similar observations were made when results were assessed as forearm blood flow.

**Figure 2:**
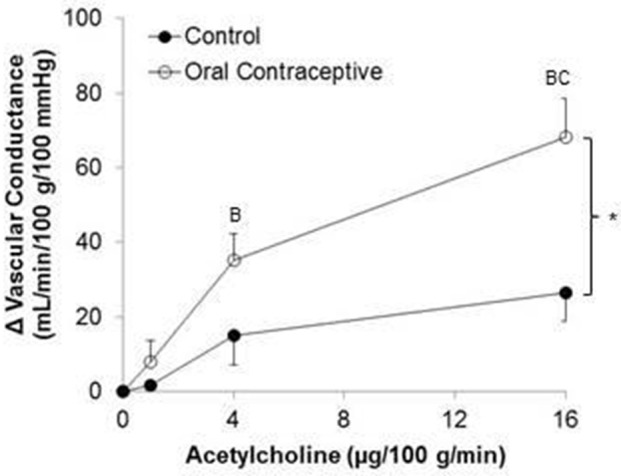
**Effect of Acetylcholine on forearm vascular conductance in women using oral contraceptives**. (Control, *n* = 6; Oral contraceptive, *n* = 8) Acetylcholine infusion resulted in a dose-dependent increase in vascular conductance (*p* < 0.001). The rise in vascular conductance from baseline with Acetylcholine infusion was greater in women taking oral contraceptives when compared to controls (*p* = 0.006). Data are presented as Mean ± SEM. ^*^p < 0.05 vs. Control; ^B^*p* < 0.05 vs. 1 μg/100 g/min; ^C^*p* < 0.05 vs. 4 μg/100 g/min.

Acetylcholine was infused in a sub-set of subjects (Control, *n* = 6; Oral Contraceptive, *n* = 8). Acetylcholine infusion resulted in a dose-dependent increase in vascular conductance (Table 3, Figure 2; Main effect of ACH *p* < 0.001). Acetylcholine-mediated vasodilation was greater in women taking oral contraceptives when compared to control women (Figure 2; Main effect of group, *p* = 0.007). Similarly, the rise (Δ) in vascular conductance from baseline with Acetylcholine infusion was greater in women taking oral contraceptives when compared to controls (Figure 2; Main effect of group, *p* = 0.006). No group-by-dose interactions were observed. Similar observations were made when results were assessed as forearm blood flow.

**Table 3:** **Effect of Acetylcholine in women using oral contraceptives**.

	**Control (*n* = 6)**	**Oral Contraceptive (*n* = 8)**	**Main effect of Acetylcholine**	**Main effect of Group**
**Diameter (cm)**				
Baseline	0.35 ± 0.02	0.34 ± 0.01	0.090	0.582
1 μg/100 g/min	0.34 ± 0.02	0.35 ± 0.01		
4 μg/100 g/min	0.36 ± 0.02	0.37 ± 0.01		
16 μg/100 g/min	0.37 ± 0.02	0.38 ± 0.01		
**Heart Rate (beats/min)**				
Baseline	54 ± 3	65 ± 3	0.983	**<0.001**
1 μg/100 g/min	55 ± 3	66 ± 3		
4 μg/100 g/min	54 ± 3	64 ± 2		
16 μg/100 g/min	56 ± 4	64 ± 2		
**Mean Arterial Blood Pressure (mmHg)**				
Baseline	83 ± 4	85 ± 3	0.843	0.905
1 μg/100 g/min	83 ± 3	84 ± 3		
4 μg/100 g/min	81 ± 4	81 ± 3		
16 μg/100 g/min	83 ± 5	81 ± 3		
**Forearm Blood Flow (mL/min/100 g)**				
Baseline	7.0 ± 1.0	6.8 ± 0.8	**<0.001**	**0.004**
1 μg/100 g/min	8.7 ± 2.6	13.2 ± 4.7		
4 μg/100 g/min	21.9 ± 5.7^A^^B^	35.5 ± 6.3^A^^B^		
16 μg/100 g/min	33.3 ± 5.1^A^^B^^C^	62.0 ± 8.8^A^^B^^C^		
**Forearm Vascular Conductance (mL/min/100 g/100 mmHg)**				
Baseline	8.2 ± 0.9	8.1 ± 1.1	**<0.001**	**0.007**
1 μg/100 g/min	10.5 ± 3.4	16.0 ± 5.9		
4 μg/100 g/min	27.9 ± 7.8^A^^B^	43.2 ± 7.6^A^^B^		
16 μg/100 g/min	41.9 ± 7.4^A^^B^^C^	76.2 ± 10.8^A^^B^^C^		

*Data are presented as Mean ± SEM*.

A p < 0.05 vs. Baseline;

B p < 0.05 vs. 1 μg/100 g/min;

C *p < 0.05 vs. 4 μg/100 g/min. From the initial model which included Group-by-Acetylcholine interaction effect, no significant interaction was detected (p > 0.05). Bold values highlight significance*.

**Figure 3:**
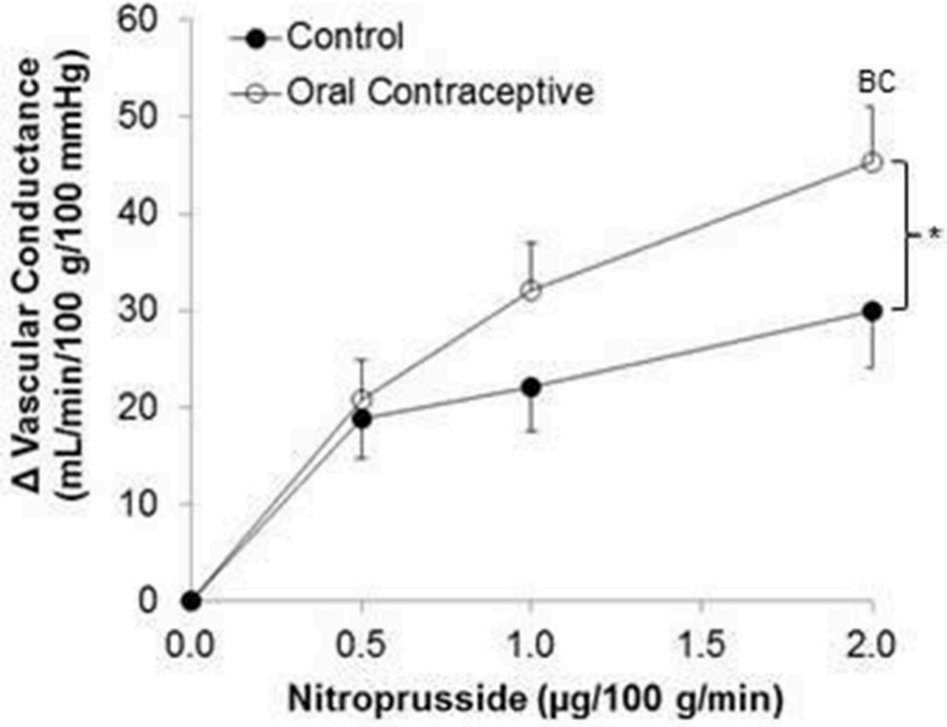
**Effect of Nitroprusside on forearm vascular conductance in women using oral contraceptives**. (Control, *n* = 10; Oral contraceptive, *n* = 13) Nitroprusside infusion resulted in a dose-dependent increase in vascular conductance. The rise in vascular conductance from baseline with Nitroprusside infusion was greater in women taking oral contraceptives when compared to controls (*p* = 0.032). Data are presented as Mean±SEM. ^*^p < 0.05 vs. Control; ^B^*p* < 0.05 vs. 0.05 μg/100 g/min; ^C^*p* < 0.05 vs/ 1.0 μg/100 g/min.

Nitroprusside infusion (Control, *n* = 10; Oral contraceptive, *n* = 13) resulted in a dose-dependent increase in vascular conductance (Table 4, Figure 3; Main effect of NTP *p* < 0.001). Nitroprusside-mediated vasodilation was greater in women taking oral contraceptives when compared to control women (Figure 3; Main effect of group, *p* = 0.015). Similarly, the rise (Δ) in vascular conductance from baseline with Nitroprusside infusion was greater in women taking oral contraceptives when compared to controls (Figure 3; Main effect of group, *p* = 0.032). No group-by-dose interactions were observed. Similar observations were made when results were assessed as forearm blood flow.

**Table 4:** **Effect of Nitroprusside in women using oral contraceptives**.

	**Control (*n* = 10)**	**Oral Contraceptive (*n* = 13)**	**Main effect of Nitroprusside**	**Main effect of Group**
**Diameter (cm)**				
Baseline	0.34 ± 0.01	0.33 ± 0.01	**<0.001**	0.072
0.5 μg/100 g/min	0.35 ± 0.01^A^	0.35 ± 0.01^A^		
1.0 μg/100 g/min	0.37 ± 0.01^A^	0.36 ± 0.01^A^		
2.0 μg/100 g/min	0.39 ± 0.01^A^^B^^C^	0.38 ± 0.01^A^^B^^C^	
**Heart Rate (beats/min)**				
Baseline	59 ± 3	65 ± 3	0.329	**<0.001**
0.5 μg/100 g/min	57 ± 2	68 ± 4		
1.0 μg/100 g/min	60 ± 2	71 ± 3		
2.0 μg/100 g/min	62 ± 3	72 ± 3		
**Mean Arterial Blood Pressure (mmHg)**				
Baseline	86 ± 3	90 ± 2	**0.009**	**0.039**
0.5 μg/100 g/min	79 ± 3^A^	85 ± 3^A^		
1.0 μg/100 g/min	79 ± 3^A^	82 ± 2^A^		
2.0 μg/100 g/min	78 ± 2^A^	80 ± 3^A^		
**Forearm Blood Flow (mL/min/100 g)**				
Baseline	5.8 ± 0.9	6.9 ± 0.7	**<0.001**	**0.004**
0.5 μg/100 g/min	20.3 ± 3.5^A^	24.0 ± 2.7^A^		
1.0 μg/100 g/min	22.8 ± 3.9^A^	32.2 ± 3.5^A^		
2.0 μg/100 g/min	28.6 ± 5.0^A^^B^^C^	40.7 ± 3.1^A^^B^^C^		
**Forearm Vascular Conductance (mL/min/100 g/100 mmHg)**				
Baseline	6.7 ± 0.9	7.7 ± 0.8	**<0.001**	**0.015**
0.5 μg/100 g/min	25.6 ± 4.3^A^	28.6 ± 3.8^A^		
1.0 μg/100 g/min	28.9 ± 4.9^A^	39.7 ± 4.9^A^		
2.0 μg/100 g/min	36.7 ± 6.2^A^^B^^C^	53.0 ± 5.7^A^^B^^C^		

*Data are presented as Mean ± SEM*.

A p < 0.05 vs. Baseline;

B p < 0.05 vs. 0.5 μg/100 g/min;

C *p < 0.05 vs. 1.0 μg/100 g/min. From the initial model which included Group-by-Nitroprusside interaction effect, no significant interaction was detected (p > 0.05). Bold values highlight significance*.

## Discussion

This is the first study to directly test the hypothesis that oral contraceptive use increases β-adrenergic vasodilation. Novel findings from the present study show that Isoproterenol-mediated vasodilation in the forearm is higher in young women currently using oral contraceptives when compared to women with natural menstrual cycles studied during the placebo pill/early follicular phase (Table 2, Figure 1).

There are several mechanisms by which oral contraceptive use may increase β-adrenergic receptor mediated vasodilation. First, although doses of estradiol used in oral contraceptive pills are considered very low, they are physiologically higher than naturally circulating sex hormones. Estrogen can increase nitric oxide availability (Kleinert et al., 1998; Cicinelli et al., 1999) and, although controversy exists (Limberg et al., 2016), it is thought that β-adrenergic receptors cause vasodilation partially through a nitric oxide mechanism (Cardillo et al., 1997; Ferro et al., 1999; Kneale et al., 2000; Jordan et al., 2001). Second, oral contraceptives can suppress androgen levels (Wiegratz et al., 1995; Coenen et al., 1996) and androgen suppression has been shown to increase endothelium-dependent vasodilation (Herman et al., 1997). Third, estrogen may also have a direct effect on β-adrenergic receptors through an endothelium-independent mechanism (Ferrer et al., 1996). With these ideas in mind, we explored potential differences in smooth muscle responsiveness to exogenous nitric oxide (Nitroprusside) and/or endothelium-dependent vasodilation (Acetylcholine) between women currently using oral contraceptives and women with natural menstrual cycles.

We observed higher Acetylcholine-mediated vasodilation in the women using oral contraceptive pills when compared to women with natural menstrual cycles (Figure 2). In contrast, Virdis and colleagues reported that 6-months of hormonal contraceptive use did not affect microcirculatory responses to Acetylcholine (Virdis et al., 2003). Additionally, John and colleagues found similar Acetylcholine-mediated vasodilation between women using oral contraception versus those who were not currently using oral contraceptive pills (John et al., 2000). However, both studies focused on women during the follicular phase of the menstrual cycle (~day 12), characterized by high serum estradiol levels (John et al., 2000; Virdis et al., 2003). With our new findings, it appears that improvements in endothelial function with oral contraceptive use may be similar in magnitude to increased function observed during the follicular phase of the menstrual cycle in normally cycling women.

Vasodilatory responses to exogenous nitric oxide administration (Nitroprusside) have been shown to be similar between women using oral contraceptive pills and those with natural menstrual cycles (John et al., 2000; Virdis et al., 2003). In contrast, we found that women using oral contraceptives demonstrated greater Nitroprusside responses (Figure 3). As noted above, differences in study design (studying women on days 1–5 vs. day 12 of the menstrual cycle) may have contributed to discrepancies between findings. On the other hand, L-NMMA (a nitric oxide synthase inhibitor) reduced basal forearm blood flow more in women using oral contraception when compared to those without (John et al., 2000). Taken together, it is reasonable to propose: (1) the vascular smooth muscle is more responsive to exogenous nitric oxide in women currently using oral contraceptives (Figure 3), (2) oral contraceptive use increases nitric oxide production and/or release (John et al., 2000), and/or (3) any increase in responsiveness to nitric oxide may be due to changes in female sex hormone levels (John et al., 2000). Future studies using nitric oxide synthase inhibition will be necessary to adequately address such possibilities.

## Clinical implications

Approximately 80% of women in the United States will use oral contraceptive pills during their lifetime (Shufelt and Bairey Merz, 2009; Boldo and White, 2011; Maguire and Westhoff, 2011). Present novel findings indicate oral contraceptive use produces a broad, positive impact on peripheral skeletal muscle microvascular function, including greater β-adrenergic receptor mediated vasodilation, as well as endothelium-dependent and -independent vasodilation that is observed even during the placebo pill phase. Consistent with greater vasodilation, women using oral contraceptives appear to exhibit less sympathetic support of blood pressure (Harvey et al., 2015). Many have speculated this effect to be due to greater β-adrenergic receptor mediated vasodilation (Hart et al., 2011; Joyner et al., 2016) and our data directly support this hypothesis. With this in mind, it may be surprising that the women currently using oral contraceptives exhibited mildly elevated blood pressures (~2–4 mmHg) when compared with control women (Tables 2–4). Oral contraceptive use has been associated with increased blood pressure (Narkiewicz et al., 1995; Cardoso et al., 1997; Boldo and White, 2011; Brito et al., 2011; Maguire and Westhoff, 2011) independent of changes in muscle sympathetic nervous system activity (Harvey et al., 2015). Although exact mechanisms are unknown, this may be the result of increased plasma volume (Stachenfeld and Taylor, 2005; Cherney et al., 2007) and/or increased activity of the renin-angiotensin-aldosterone system (Gordon et al., 1992; Oelkers, 1996; Kang et al., 2001). Therefore, despite the positive impact of oral contraceptive use on peripheral vascular function—as suggested by the present findings—combined effects of oral contraceptives on other physiological control mechanisms likely attenuate this “protective effect” when systemic blood pressure is examined. However, any effect is likely cyclical, given blood pressure is often lower during the active phase of oral contraceptive use when compared to the placebo phase (Meendering et al., 2010).

## Experimental considerations

This is the first study to examine β-adrenergic receptor mediated vasodilation in women using oral contraceptives and potential contributing mechanisms. Despite this, there are some important experimental considerations. First, this study is a *post-hoc* analysis and therefore women were taking different formulations of oral contraceptive pills. However, the majority (70%) of participants were known to be using third- and fourth-generation formulations (Supplemental Table S1), which have been shown to have beneficial, rather than detrimental, effects on endothelial function (Simoncini et al., 2007; Meendering et al., 2010) and this is reflected in observed increases in endothelium-dependent vasodilation. Second, the duration of oral contraceptive use is unknown; however, duration of contraceptive use has been shown previously to have little-to-no effect on vascular function (Friedman et al., 2011). Third, women taking oral contraceptives had significantly higher body fat percentage, blood pressure, and total cholesterol (Tables 1–2). Consistent with this, changes in lipid profile have been shown previously with oral contraceptive use (Sitruk-Ware, 2006; Shufelt and Bairey Merz, 2009). However, these factors should contribute to lower endothelium-dependent vasodilation, rather than higher (Virdis et al., 2003) and therefore would likely lead us to underestimate group differences. Lastly, we did not measure sex hormone levels. However, based on previous literature, including data from our laboratory (Peltonen et al., 2015), we are confident endogenous female sex hormones were low in both groups when studied during the early follicular/placebo phases (Aden et al., 1998). Despite relatively low hormone levels in both groups, significant differences in β-adrenergic mediated vasodilation were observed. These data may support the possibility of a carry-over effect from the prior active pill phase, although future studies will be necessary to examine this possibility.

## Conclusion

This is the first study to directly test the hypothesis that oral contraceptive use increases β-adrenergic receptor mediated vasodilation in the forearm skeletal muscle microcirculation of young, premenopausal women. Present novel findings show greater forearm β-adrenergic receptor mediated vasodilation, as well as endothelium-dependent and -independent vasodilation, in women currently using oral contraceptives when studied during the placebo phase. Taken together, these data provide important new insight into the effect of oral contraceptive use on vascular function, which may have important implications for neurovascular control in both health and disease.

## Author contributions

WS provided oversight; JL and WS designed study; JL, GP, RJ, JH, JK, ME, JS, BW, and WS collected, analyzed, and interpreted data; JL, GP, and WS drafted manuscript; JL, GP, RJ, JH, JK, ME, JS, BW, and WS revised manuscript.

## Funding

NIH HL 105820 (WS), NIH F32 HL120570 (JL).

### Conflict of interest statement

The authors declare that the research was conducted in the absence of any commercial or financial relationships that could be construed as a potential conflict of interest.
