# Supplementary material for: Greater Beta-Adrenergic Receptor Mediated Vasodilation in Women Using Oral Contraceptives
Source: Front Physiol. 2016 Jun 8;7:215. doi: 10.3389/fphys.2016.00215 (PMC4896959; doi:10.3389/fphys.2016.00215)
Supplement: Supplementary file 1 [file Table1.docx]

| **N** | **Brand (s)** | **Type** | **Generation** | **Phase** | **Days** | **Estrogen (dose)** | **Progestin (dose)** |
| --- | --- | --- | --- | --- | --- | --- | --- |
| 2 | Microgestin Fe,  Junel Fe | Monophasic | 1 | 0 | 1-7 | -- | -- |
|  |  |  |  | 1 | 8-28 | Ethinyl Estradiol (0.020 mg) | Norethindrone (1.000 mg) |
| 2 | Yaz | Monophasic | 4 | 0 | 1-4 | -- | -- |
|  |  |  |  | 1 | 5-28 | Ethinyl Estradiol (0.020 mg) | Drospirenone (3.000 mg) |
| 3 | Reclipsen,  Desogen, Apri | Monophasic | 3 | 0 | 1-7 | -- | -- |
|  |  |  |  | 1 | 8-28 | Ethinyl Estradiol (0.030 mg) | Desogestrel (0.150 mg) |
| 1 | Kariva | Biphasic | 3 | 0 | 1-2 | -- | -- |
|  |  |  |  | 1 | 3-7 | Ethinyl Estradiol (0.010 mg) | -- |
|  |  |  |  | 2 | 8-28 | Ethinyl Estradiol (0.020 mg) | Desogestrel (0.150 mg) |
| 3 | Tri-Sprintec,  Tri-Linyah,  Trinessa 28 | Triphasic | 3 | 0 | 1-7 | -- | -- |
|  |  |  |  | 1 | 8-14 | Ethinyl Estradiol (0.035 mg) | Norgestimate (0.180 mg) |
|  |  |  |  | 2 | 15-21 | Ethinyl Estradiol (0.035 mg) | Norgestimate (0.215 mg) |
|  |  |  |  | 3 | 22-28 | Ethinyl Estradiol (0.035 mg) | Norgestimate (0.250 mg) |
| 2 | Unknown | -- | -- | -- | -- | -- | -- |

**Supplemental Table S1: Classification of oral contraception among users**
